# A Novel TORC1 Inhibitor, RLX‐201, Improves Skin Health, Biomechanics, and Extracellular Matrix Integrity in a Double‐Blind Clinical Study

**DOI:** 10.1111/jocd.71219

**Published:** 2026-10-07

**Authors:** Zoe D. Draelos, Rahul C. Mehta, Manisha Jha, Doris Day, Manpreet K. Randhawa

**Affiliations:** ^1^ Dermatology Consulting Services High Point, Durham North Carolina USA; ^2^ Rapalogix Health Inc. New York New York USA; ^3^ Department of Dermatology Day Dermatology and Aesthetics New York New York USA

**Keywords:** hallmarks of aging, mTOR, photoaging, skin longevity, TORC1

## Abstract

**Background:**

Skin aging is characterized by decline in structural integrity, extracellular matrix (ECM) organization, and biomechanical function. Emerging concepts in skin longevity emphasize preservation of tissue resilience through modulation of aging pathways, including mechanistic target of rapamycin complex 1 (TORC1), a central regulator of the 12 currently accepted hallmarks of aging.

**Aim:**

Evaluate the effect of topical formulations containing RLX‐201 on clinical photoaging, skin biomechanics, and molecular biomarkers.

**Methods:**

In this 12‐week, randomized, double‐blind, vehicle‐controlled study, female subjects with moderate‐to‐severe facial photoaging applied RLX‐201 formulation or vehicle twice daily. Clinical assessments were performed at baseline and Weeks 2, 4, 8, and 12 by an investigator. Skin viscoelastic properties were quantified using elastometry. Subject self‐assessments were collected. Mechanistic evaluation was conducted in 3D full‐thickness skin tissue models, assessing phosphorylation of ribosomal protein S6 (pRPS6) as a surrogate marker of mTORC1 activity.

**Results:**

Treatment with RLX‐201 resulted in significant improvements in clinical parameters of skin firmness, elasticity, sagging, and overall skin quality compared with vehicle (*p* ≤ 0.05), with onset observed as early as Week 2 and sustained through Week 12. Biomechanical analysis demonstrated significant enhancement in viscoelastic properties. Subject‐reported outcomes showed high concordance with investigator assessments, with > 85% of participants reporting improvements in radiance, texture, and hydration. Tissue analysis demonstrated suppression of TORC1 activity, with ~91% reduction in pRPS6 expression.

**Conclusions:**

Topical modulation of TORC1 improves clinical, biomechanical, and molecular features of skin aging. These findings support a mechanistic approach to enhancing skin health, in which targeting nutrient‐sensing pathways enhances tissue resilience and functional performance, extending beyond conventional cosmetic outcomes.

## 1 Introduction

Skin longevity represents an emerging paradigm in aesthetics; one that focuses on preserving biological resilience and functional performance of skin across time rather than simply reversing visible signs of aging [1]. Although there is no formal definition, the concept of skin longevity parallels that of cellular or tissue resiliency: the capacity of skin to maintain its structure, ability to repair, and preserve its functional integrity, despite both internal and external stressors. In this context, maintaining the skin's natural ability to renew and repair is fundamental to long‐term skin health and function [2, 3]. Although most longevity research focuses on internal organs, the skin represents a uniquely accessible human tissue in which age‐associated biological decline can be measured both molecularly and biomechanically in real time.

Cutaneous aging recapitulates core systemic hallmarks. Dermal fibroblasts exhibit reduced metabolic efficiency, impaired autophagic flux, accumulation of damaged proteins, mitochondrial dysfunction, and increased senescence burden [4, 5, 6]. These intracellular changes manifest extracellularly as fragmentation of elastin fibers, decreased collagen synthesis, disorganized fibrillar architecture, and reduced tensile strength [7]. Thus, the extracellular matrix (ECM) functions not merely as structural scaffolding, but as a systems level readout of cellular proteostasis and regenerative capacity. Within this framework, nutrient‐sensing pathways particularly mechanistic target of rapamycin complex 1 (TORC1) play a central role in determining tissue longevity. mTORC1 integrates growth factor, nutrient, and energy signals to regulate anabolic processes and autophagy [8, 9]. While transient activation supports growth and repair, chronic mTORC1 hyperactivation observed in aged tissues suppresses autophagy, disrupts proteostasis, enhances oxidative stress, and accelerates cellular senescence [10, 11]. Genetic and pharmacologic attenuation of TORC1 signaling has been repeatedly shown to extend lifespan and healthspan across model organisms.

In human skin, dysregulated TORC1 signaling has been linked to impaired fibroblast renewal, reduced ECM synthesis, altered barrier homeostasis, and diminished regenerative potential [12]. These alterations converge to reduce mechanical resilience, reflected clinically as decreased elasticity and firmness, biomechanical endpoints that serve as functional biomarkers of tissue aging. Elasticity represents the integrated outcome of ECM organization, fibroblast functionality, and effective cellular maintenance systems. Therefore, interventions capable of restoring biomechanical performance may reflect restoration of upstream cellular longevity pathways, rather than superficial structural effects alone.

RLX‐201 is a selective topical modulator of TORC1 designed to recalibrate nutrient‐sensing activity in aged skin. Preclinical data indicate that RLX‐201 selectively suppresses mTORC1 signaling while preserving essential anabolic balance, thereby potentially supporting proteostatic maintenance and ECM integrity [13]. The present study evaluates whether selective TORC1 modulation translates into measurable improvements in biomechanical function and molecular markers of aging in human skin.

By positioning elasticity and mechanical recovery as functional readouts of cellular resilience, this study aims to test whether targeted nutrient‐sensing modulation can promote tissue‐level rejuvenation in a clinically measurable manner.

## 2 Materials and Methods

### 2.1 Study Design and Participants

This was a 12‐week, single‐center, double‐blind, randomized, vehicle‐controlled, parallel‐group clinical study designed to evaluate the efficacy, safety, and tolerability of a topical selective mechanistic target of rapamycin complex 1 (TORC1) inhibitor formulation, RLX‐201 (Re‐Q Pro‐Longevity Face Serum), in women with moderate‐to‐severe facial photoaging. The study was conducted from August through November 2025 at Dermatology Consulting Services (High Point, NC, USA; Principal Investigator: Zoe D. Draelos, MD).

The study protocol was approved by the Allendale Institutional Review Board (AIRB; Old Lyme, CT, USA) and was conducted in accordance with the Declaration of Helsinki and Good Clinical Practice guidelines. Written informed consent was obtained from all participants before enrollment.

Healthy women aged 35–65 years with Fitzpatrick skin types I–VI and moderate‐to‐severe facial photoaging, defined as a Griffiths 10‐point scale score of 4–7, were eligible. Subjects were excluded if they had used topical prescription or over‐the‐counter facial treatments within 2 weeks before enrollment, undergone facial cosmetic procedures within 6 months, or used topical retinoid‐containing products within 3 months before baseline. A 2‐week washout period was completed before initiation of study treatment. Subject demographics and baseline characteristics are summarized in Table 1.

**TABLE 1:** Baseline demographic characteristics of study subjects.

Characteristic	RLX‐201 formulation	Vehicle control
Subjects in demographic log, *n*	30	17
Age, years, mean ± SD	54.2 ± 6.3	52.6 ± 8.5
Age range, years	39.8–62.4	40.0–65.8
Female, *n* (%)	30 (100%)	17 (100%)
Fitzpatrick skin type, *n* (%)		
Type I	3 (10.0%)	4 (23.5%)
Type II	11 (36.7%)	8 (47.1%)
Type III	0 (0.0%)	1 (5.9%)
Type IV	7 (23.3%)	0 (0.0%)
Type V	8 (26.7%)	2 (11.8%)
Type VI	1 (3.3%)	2 (11.8%)
Race, *n* (%)		
Asian	0 (0.0%)	0 (0.0%)
Black/African American	15 (50.0%)	4 (23.5%)
White/Caucasian	13 (43.3%)	12 (70.6%)
Hispanic	2 (6.7%)	1 (5.9%)

### 2.2 Randomization and Treatment

Forty‐seven subjects were randomized according to a computer‐generated randomization schedule to receive either RLX‐201 (*n* = 31) or a matching vehicle control (*n* = 16). Treatment allocation was concealed using identical, unbranded packaging and labeling. Participants, investigators, outcome assessors, and study personnel remained blinded to treatment assignment throughout the study.

The assigned study formulation was applied to the entire face twice daily, once in the morning and once in the evening, for 12 weeks. In the morning, participants applied Neutrogena Ultra Gentle Daily Facial Moisturizer SPF 30 followed by the assigned study formulation. In the evening, participants applied Cetaphil moisturizing lotion followed by the assigned study formulation. Participants were instructed to avoid excessive sun exposure during the study period.

Treatment compliance was assessed using participant daily diaries and gravimetric measurement of study product containers. Study visits were conducted at baseline and Weeks 2, 4, 8, and 12.

### 2.3 Clinical and Instrumental Assessments

The primary efficacy endpoint was the change from baseline to Week 12 in investigator‐assessed facial skin firmness and elasticity compared with vehicle. Secondary and exploratory efficacy assessments included overall photoaging, skin texture, cheek wrinkles, radiance, luminosity, skin sagging, and skin tone.

Clinical assessments were performed by a blinded dermatologist at baseline and Weeks 2, 4, 8, and 12. Subject self‐assessment questionnaires and standardized clinical photography were performed at the same visits. Cutaneous tolerability was assessed at each visit by evaluating erythema, edema, dryness/desquamation, itching, burning, stinging, and irritation. Clinical grading was performed using a 5‐point ordinal scale ranging from 0 (none) to 4 (severe). Adverse events were recorded throughout the study.

Instrumental assessments included Cutometry for skin elasticity. Standardized facial imaging was performed on clean, product‐free skin using the VISIA CR 4.3 imaging system (Canfield Scientific Inc., Parsippany, NJ, USA). Frontal and bilateral 45° facial images were obtained at each evaluation visit.

### 2.4 Exploratory Biopsy

Twelve punch biopsies were obtained from the postauricular region of six participants for exploratory histologic and molecular analyses. Biopsy sites were cleansed with alcohol and anesthetized with 2% xylocaine with epinephrine. Samples were collected into 10% neutral buffered formalin for subsequent paraffin embedding and histologic evaluation.

### 2.5 Statistical Analysis

The Week 12 assessment was prespecified as the primary efficacy time point, with assessments at Weeks 2, 4, and 8 considered secondary time points. Secondary efficacy outcomes included investigator‐assessed skin quality and photoaging parameters, instrumental elasticity measurements, and subject self‐assessment outcomes. Histologic and molecular assessments were considered exploratory.

Continuous variables were summarized using descriptive statistics and analyzed using Student's *t*‐test. Ordinal clinical grading outcomes were analyzed as changes from baseline using the Wilcoxon signed‐rank test. Statistical significance was defined as *p* ≤ 0.05.

All participants who provided informed consent and received study treatment were included in the safety population. Efficacy analyses used all available data collected through the time of discontinuation. Forty‐four of the 47 enrolled participants completed the 12‐week study. Three participants discontinued and were lost to follow‐up for personal reasons; data collected before discontinuation were retained in the analyses.

Analyses were performed at each scheduled evaluation time point, with primary emphasis on Week 12 and comparisons between the RLX‐201 and vehicle groups. Results were summarized using mean changes from baseline, percentage improvement, *p*‐values, and the number of participants contributing data to each analysis.

### 2.6 Bioinstrumentation Measurements

Cutaneous elasticity was quantified using the DermaLab Elasticity probe (Cortex Technology, Aalborg, Denmark), a high‐precision suction‐based elastometer. The device applied a programmed negative pressure to induce vertical skin deformation, which was monitored in real‐time by a centrally positioned infrared displacement sensor. The resulting stress–strain data were processed to determine the Young's Elastic Modulus, a direct measure of tissue stiffness, and the Retraction Time, representing the speed of elastic recovery [14]. These biomechanical endpoints were utilized as quantitative indicators of the structural health of the dermal extracellular matrix and the skin's regenerative response to the treatment protocol, as established in skin aging studies.

### 2.7 Western Blot Analysis of mTORC1 Activity in Full‐Thickness Human Skin Models

Phosphorylated ribosomal protein S6 (pRPS6), a downstream marker of mechanistic target of rapamycin complex 1 (TORC1) activity, was evaluated in full‐thickness human skin models (MatTek Corporation, Ashland, MA, USA) obtained from three independent donors. Skin models were treated with RLX‐201‐containing formulations or water controls according to the experimental design.

TORC1 activity was quantified by automated capillary‐based Western blotting using the Wes system (ProteinSimple, San Jose, CA, USA). Total protein was extracted from treated skin tissue and quantified using the Pierce BCA Protein Assay Kit (cat. no. 23227; Thermo Fisher Scientific, Waltham, MA, USA), with bovine serum albumin (BSA) as the protein standard. Absorbance was measured using a SpectraMax i3x multimode microplate reader (Molecular Devices, San Jose, CA, USA).

Protein samples, molecular weight standards, primary and secondary antibodies, and chemiluminescent detection reagents were loaded onto a 25‐capillary Wes plate using the 12–230 kDa separation module. Automated electrophoretic separation, protein immobilization, immunodetection, and chemiluminescent signal acquisition were performed according to the manufacturer's instructions. pRPS6 was detected using an anti‐phospho‐RPS6 primary antibody (R&D Systems/Bio‐Techne), followed by an HRP‐conjugated anti‐rabbit secondary antibody (ProteinSimple/Bio‐Techne). Chemiluminescent signals were quantified using Compass software (ProteinSimple) and expressed as arbitrary units (AU).

Because baseline pRPS6 expression varied among donor‐derived skin models, treatment effects were normalized to the corresponding donor‐matched water‐treated control. pRPS6 signal was normalized to total protein for each sample and expressed as a percentage change relative to the corresponding control group. Statistical comparisons between treatment and control groups were performed using Welch's *t*‐test, with *p* < 0.05 considered statistically significant.

### 2.8 Histologic Assessment of Human Skin Biopsies

Formalin‐fixed, paraffin‐embedded human skin biopsies were sectioned at approximately 4–5 μm. Sections were baked at 60°C for 90 min and deparaffinized in xylene (2 × 15 min), followed by graded ethanol (100% ethanol, 2 × 5 min; 75% ethanol, 5 min) and rehydration. Adjacent sections were stained with hematoxylin and eosin (H&E), Picrosirius Red, or elastica van Gieson (EVG) using validated laboratory protocols.

For H&E staining, sections were stained with hematoxylin and eosin to evaluate overall epidermal and dermal morphology, cellularity, and tissue integrity. Picrosirius Red staining was performed using Sirius Red solution in an acidic medium and was used to visualize and assess dermal fibrillar collagen content and organization. EVG staining was performed using a Verhoeff‐type elastic fiber stain followed by ferric chloride differentiation and Van Gieson's counterstain, allowing visualization and assessment of dermal elastic fibers.

Following staining, sections were dehydrated through graded ethanol, cleared in xylene, and coverslipped using a xylene‐based mounting medium. Slides were examined by brightfield microscopy and digitally scanned at 40× magnification using a P1000 brightfield slide scanner (3DHISTECH). Collagen and elastic fiber staining were evaluated qualitatively in standardized dermal regions using consistent imaging and analysis parameters across treatment groups.

## 3 Results

### 3.1 RLX‐201 Formulation Topical Treatment Showed Significant Clinical Improvement in Skin Firmness, Elasticity and Quality Related Skin Health Benefits as Compared to Vehicle

A total of 47 subjects were randomized (RLX‐201, *n* = 31; vehicle, *n* = 16). Forty‐four subjects completed the 12‐week study (RLX‐201, *n* = 29; vehicle, *n* = 15). Three subjects discontinued due to loss to follow‐up and were excluded from Week‐12 analyses. Efficacy analyses were conducted on subjects who completed the study. Baseline clinical grading scores and demographic characteristics were comparable between treatment groups, with no statistically significant differences observed for any efficacy parameter at baseline. Primary efficacy analyses focused on Week‐12 changes from baseline in clinical firmness and elasticity parameters. Secondary analyses evaluated earlier time points and additional skin quality attributes.

At Week 12, the active formulations containing RLX‐201, a novel mTORC1 inhibitor, showed significant improvement from baseline (*p* ≤ 0.05) in skin elasticity and quality related efficacy parameters. Differences between treatments occurred as early as Week 2, with the formulation with RLX‐201 being significantly better than vehicle (*p* ≤ 0.05) at improving firmness and continued to improve until Week 12 (Figure 1a). Skin sagging and cheek wrinkles showed significant improvement at Week 4 versus baseline, with continued improvement at Weeks 8 and 12 being significantly better than vehicle. Orbital elasticity improved significantly from Weeks 8 to 12 that was significant versus baseline and vehicle. The overall turgor of the skin directly correlated to firmness and elasticity; it also represented significant improvement from baseline from Weeks 4 to 12. The images represented improvements in respective parameters as well (Figure 1b). In particular, after 12 weeks of RLX‐201 applications, firmness and cheek wrinkles improved by 21%; sagging, orbital elasticity, and tone increased by 14% (Figure 1a).

**FIGURE 1:**
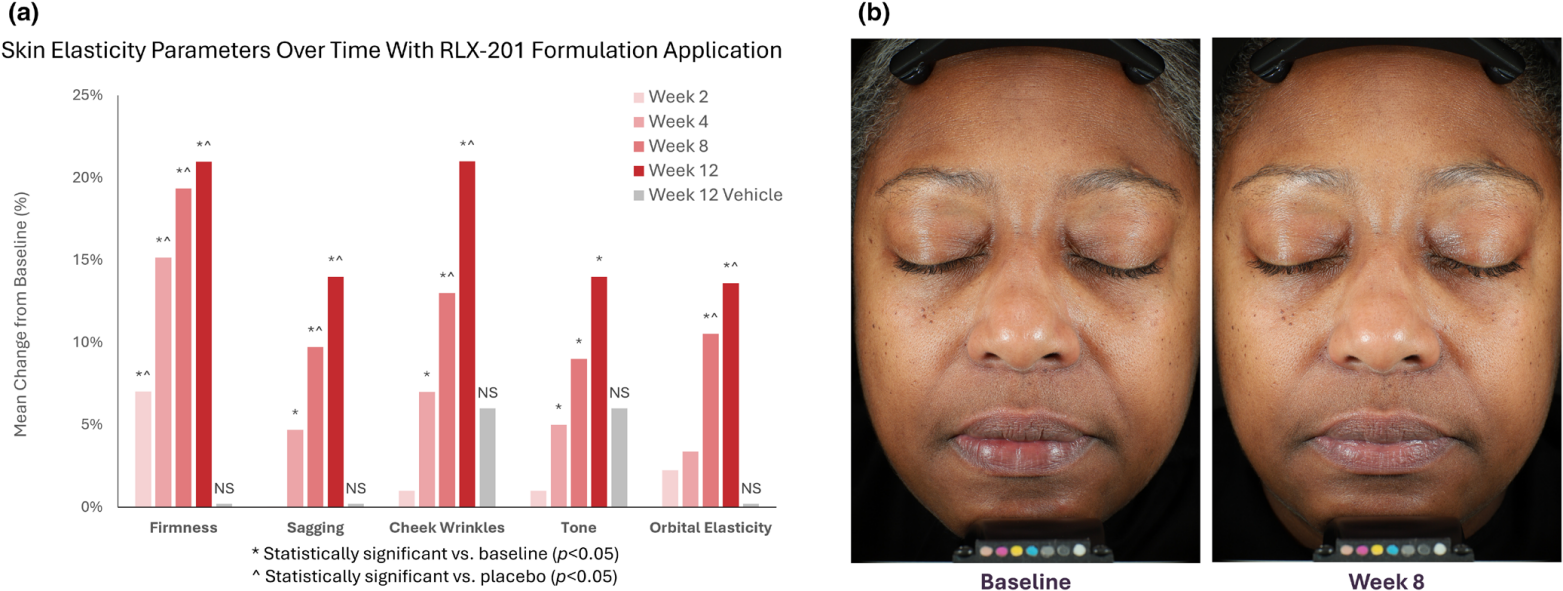
(a) RLX‐201 formulation treatment demonstrates significant improvement in skin elasticity parameters. Dermatologist graded participants at each timepoint. Participants applied RLX‐201 formulation twice daily for 12 weeks. (b) RLX‐201 formulation demonstrates improvements to the patient's skin tone, firmness, and forehead lines shown after 12 weeks. Baseline vs. Week 12 images of a participant using RLX‐201 formulation twice daily. Age 49, female, and skin type V. Imaged with Visia Standard lighting.

The skin quality parameters including radiance, luminosity, and skin texture showed significant improvement from baseline (*p* ≤ 0.05) with the active formulations containing RLX‐201. The improvement differences between treatments occurred as early as Week 2 for skin radiance and luminosity, with the formulation with RLX‐201 being significantly better than baseline (*p* ≤ 0.05) and significantly better than vehicle from Week 4, with continued improvement to Week 12. Improvement with skin texture was registered to be statistically significant versus baseline at Weeks 2 and 4, with increased improvement and statistically significant versus vehicle at Weeks 4 and 8 (Figure 2a). In particular, after 12 weeks of RLX‐201 applications, skin radiance and luminosity improved by 39% and 38%, and the skin texture improved by 40%. The images represented improvements in respective parameters as well (Figure 2b,c). These results demonstrate that the use of an RLX‐201, mTORC1‐specific inhibitor can produce continuous improvements in signs of photoaging during use.

**FIGURE 2:**
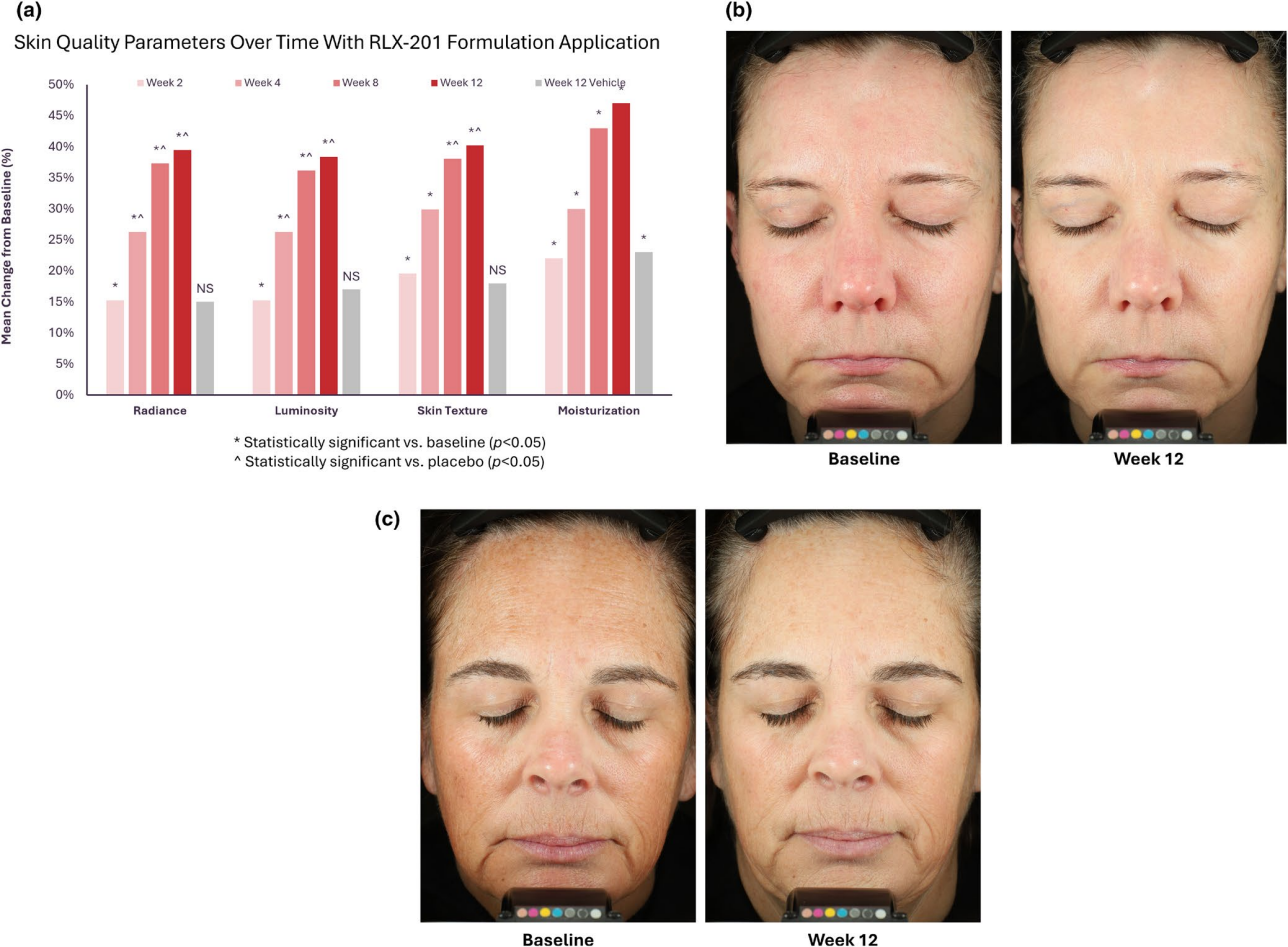
(a) RLX‐201 formulation treatment demonstrates significant improvement in skin quality parameter and PROs. Dermatologist graded participants at each timepoint. Participants applied RLX‐201 formulation twice daily for 12 weeks. (b) RLX‐201 formulation demonstrates improvements to the patient's skin redness, texture, and wrinkles shown after 12 Weeks. Baseline versus Week 12 images of a participant using RLX‐201 formulation twice a day. Age 48, female, and skin type 2. Imaged with Visia Standard lighting. (c) RLX‐201 formulation demonstrates improvements to the patient's skin firmness, skin tone, and forehead wrinkles shown after 12 weeks. Baseline versus Week 12 images of a participant using RLX‐201 formulation twice a day. Age 48, female, and skin type 2. Imaged with Visia Standard lighting.

Both formulations were well tolerated, and no adverse events were reported by any subject during the study.

### 3.2 RLX‐201 Formulation Topical Treatment Resulted in High Percentage of Agreement Between Dermatologist and Subject Self‐Perceived Assessments

Subject self‐assessments demonstrated a high level of concordance with investigator‐evaluated outcomes. At Week 12, 86% of subjects agreed that the serum visibly hydrated their skin, enhanced radiance, improved texture, and provided a nourished feel. Additionally, 72% of subjects reported that their skin appeared more youthful, 86% indicated that they would recommend the product, and 90% reported feeling optimistic about their future. While the clinical relevance of optimism is uncertain, this outcome should be considered an exploratory patient‐reported measure. Overall, the findings indicate high levels of perceived efficacy and treatment satisfaction (Table 2).

**TABLE 2:** Twice daily application of RLX‐201 formulation for 12‐weeks resulted subjects citing self‐perceived improvements in skin's condition and appearance.

% Subjects who agreed with the following self‐agreement questions	
The serum visibly hydrates my skin	86%
The serum boosts my skin radiance	86%
I see improvement in skin texture	86%
My skin feels nourished	86%
My skin looks more youthful	72%
I have been feeling optimistic about my future	90%
I would recommend the product	86%

*Note:* Twice daily application of formulation with RLX‐201 for 12‐weeks resulted in > 80% of subjects citing self‐perceived improvements in skin's condition and appearance that paralleled the grading by a dermatologist.

### 3.3 RLX‐201 Formulation Topical Treatment Showed Significant Clinical Improvement in Mechanical Properties of Skin Measured Through Elastometer

To evaluate the impact of the topical formulations on the mechanical properties of the skin, the viscoelastic component was measured using an elastometer at Weeks 2, 4, 8, and 12. The data are expressed as a mean percentage change from baseline for both the RLX‐201 formulation treatment group and the vehicle control group.

RLX‐201 formulation group demonstrated a consistent reduction in the viscoelastic component, with a decrease of approximately 9% at both Weeks 2 and 4. In contrast, the vehicle group showed an initial increase in this parameter, peaking at approximately 6% above baseline at Week 2 and remaining elevated at 4% at Week 4. The most significant divergence between the two groups occurred at Week 8. The RLX‐201 formulation treated sites exhibited the greatest improvement in skin biomechanics, reaching a peak reduction of approximately 18% from baseline. During the same period, the vehicle group returned toward baseline levels, showing a negligible change of approximately −1%. At 12‐week timepoint, the RLX‐201 formulation group maintained a robust and statistically relevant reduction in the viscoelastic component, sustaining a 13% decrease from baseline. While the vehicle group eventually showed a decrease of approximately 7% at Week 12, it remained significantly less effective than the RLX‐201 formulation throughout the study (Figure 3).

**FIGURE 3:**
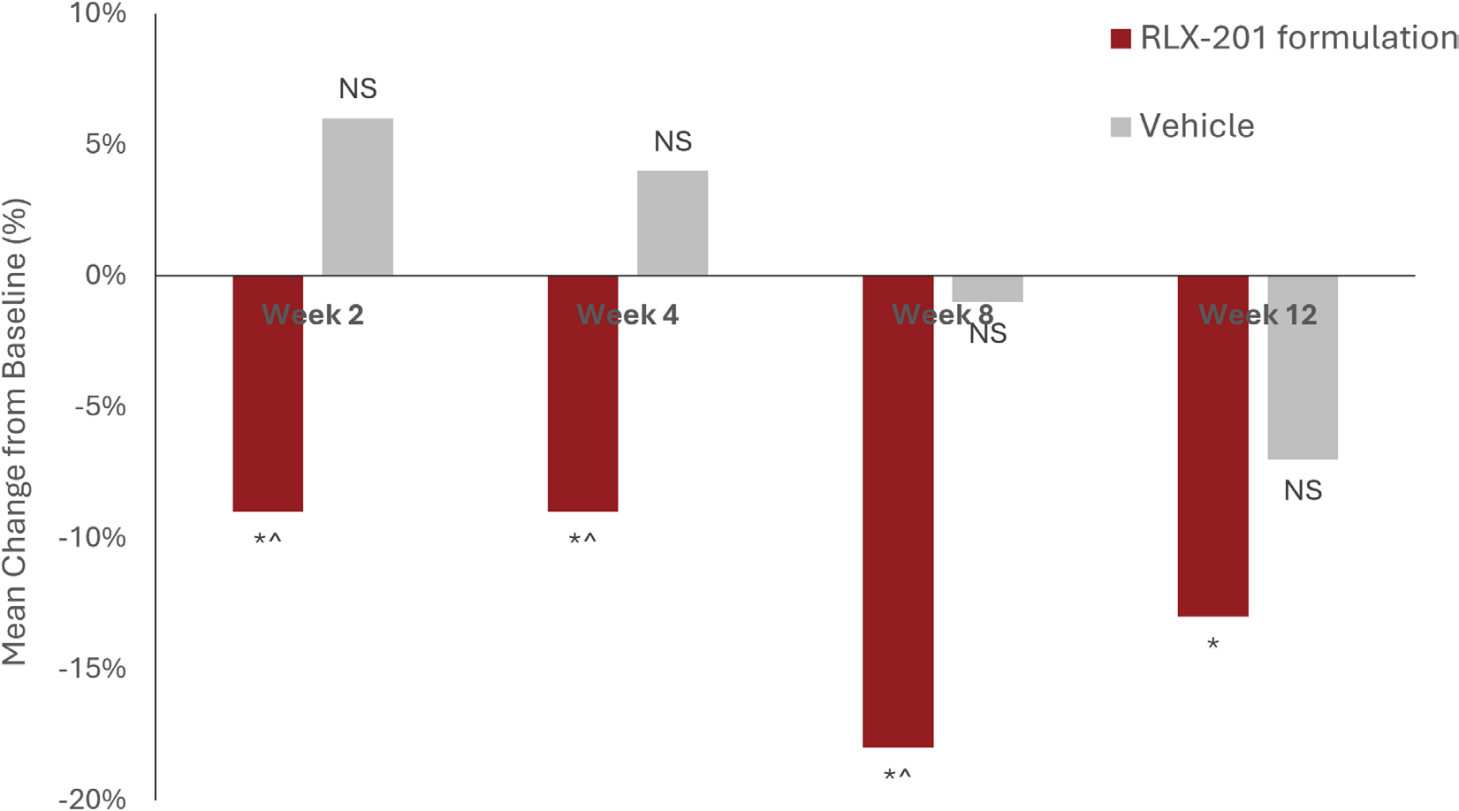
Twice daily RLX‐201 formulation application demonstrates significant improvements in skin firmness.

### 3.4 RLX‐201 Consistently Suppressed mTORC1 Activity Across Independent Full‐Thickness Human Skin Models

mTORC1 pathway activity was assessed by quantifying phosphorylated ribosomal protein S6 (pRPS6) using automated capillary‐based Western blot analysis. Substantial interdonor variability in baseline pRPS6 expression was observed among water‐treated controls, with mean values ranging from 261 568 to 4 315 735 arbitrary units (AU) across the three independent donor‐derived full‐thickness skin models, representing an approximately 16.5‐fold difference in basal pathway activity.

To account for donor‐specific differences in baseline pRPS6 expression, values from RLX‐201‐treated tissues were normalized to the corresponding donor‐matched water‐treated controls. RLX‐201 reduced pRPS6 levels to 2.7%, 17.0%, and 5.1% of control values across the three independent donor models, corresponding to reductions of 97.3%, 83.0%, and 94.9%, respectively.

Across all three donor‐derived models, mean residual pRPS6 activity was 8.3% of the corresponding water‐treated controls, representing an average reduction of 91.7% in mTORC1‐associated signaling (Figure 4). Despite substantial interdonor variability in baseline pathway activity, RLX‐201 consistently reduced pRPS6 expression across all donor models, supporting reproducible inhibition of mTORC1 pathway signaling.

**FIGURE 4:**
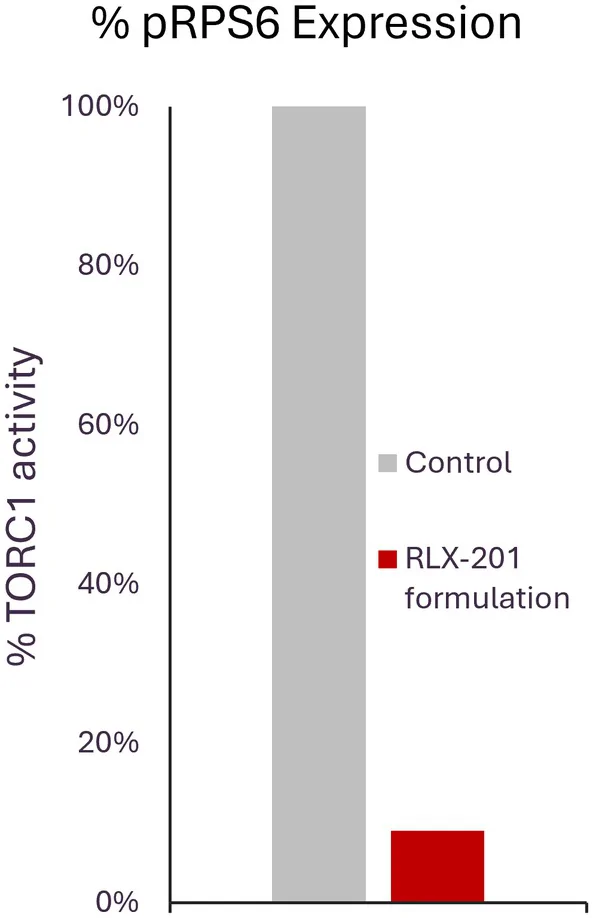
RLX201 inhibits mTORC1 activity in 3D Human Skin Model.

## 4 Discussion

This study provides translational evidence that selective modulation of mTORC1 signaling can restore measurable functional properties of aging human skin. By demonstrating improvements in elasticity, firmness, viscoelastic recovery, and structural matrix components alongside suppression of mTORC1 activity, the findings support the hypothesis that targeted nutrient‐sensing recalibration can enhance tissue‐level resilience.

Aging is characterized by progressive disruption of interconnected molecular hallmarks, including impaired proteostasis, mitochondrial dysfunction, cellular senescence, and dysregulated nutrient sensing. mTORC1 occupies a central regulatory node within this network. Chronic hyperactivation of mTORC1, observed in aged tissues, suppresses autophagy, disturbs protein quality control, increases oxidative stress, and reinforces senescence‐associated phenotypes [15]. In dermal fibroblasts, these effects translate into reduced collagen synthesis, elastin fragmentation, and compromised extracellular matrix (ECM) organization, key drivers of diminished biomechanical performance [16].

In the present study, topical RLX‐201 significantly reduced phosphorylation of pRPS6 in human 3D skin models, confirming effective attenuation of mTORC1 signaling. The magnitude of pRPS6 suppression (~91%) indicates robust pathway engagement and supports a biologically meaningful recalibration of nutrient‐sensing activity (Figure 4).

The clinical and biomechanical data align with this molecular modulation. Quantitative elastometry demonstrated sustained reductions in viscoelastic deformation, with peak improvement at Week 8 and maintenance through Week 12. Improvements in firmness, sagging, and orbital elasticity were statistically significant and progressive. Elasticity is an integrative biomarker reflecting ECM organization, fibroblast competence, and effective intracellular repair systems. Therefore, the observed enhancement in mechanical resilience likely reflects structural remodeling rather than transient surface tightening.

Histological analysis further supports this interpretation. RLX‐201 treatment was associated with increased elastin and collagen synthesis, providing structural evidence of matrix reinforcement (Figure 5a–c). Enhanced deposition and organization of these ECM components offer a mechanistic bridge between mTORC1 modulation and improved biomechanical performance. Restoration of elastin integrity and collagen architecture directly contributes to tensile strength, recoil efficiency, and resistance to mechanical fatigue, core determinants of tissue healthspan.

**FIGURE 5:**
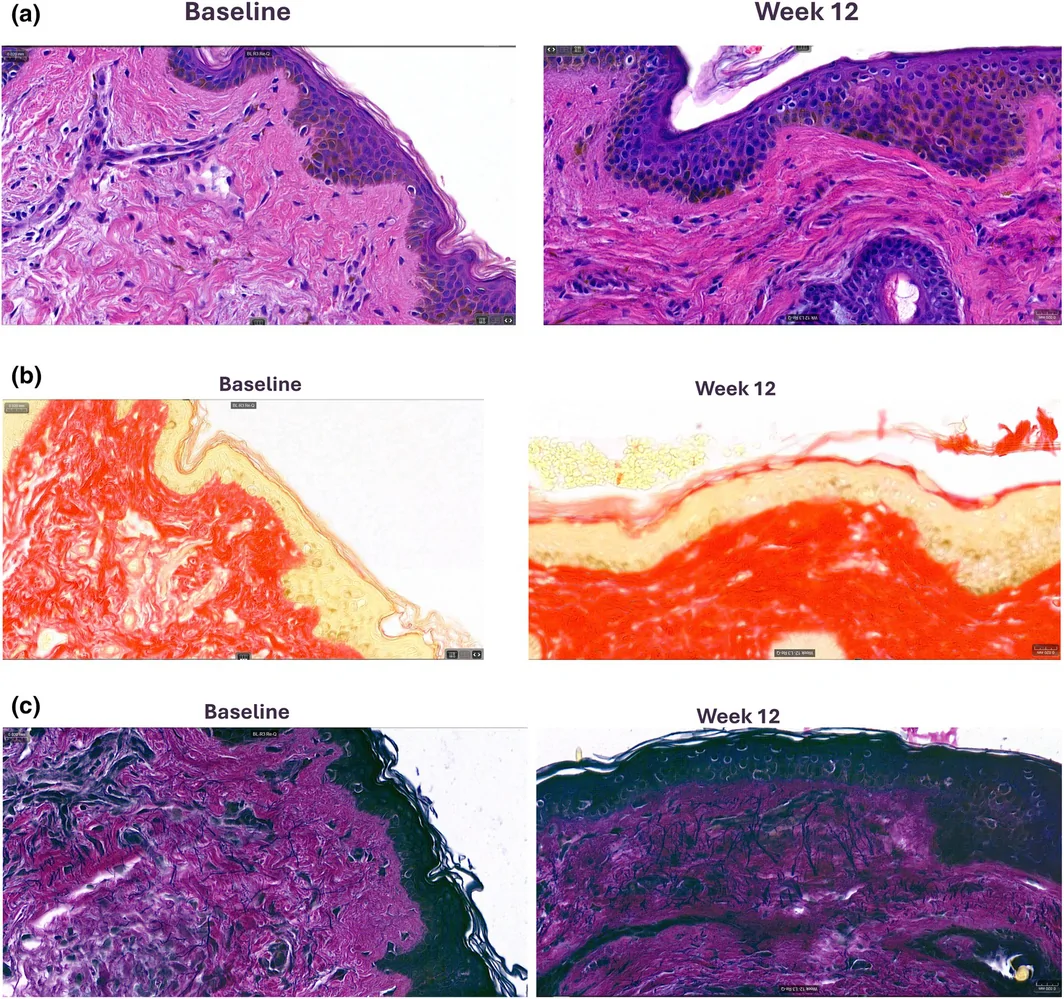
(a) Participant histology on biopsies from baseline and week 12 after using RLX‐201 formulation twice daily. Hematoxylin and Eosin‐stained dermal tissue at 40× magnification. (b) Participant histology on biopsies from baseline and Week 12 after using RLX‐201 formulation twice daily. Sirius Red‐stained dermal tissue at 40× magnification. (c) Participant histology on biopsies from baseline and Week 12 after using RLX‐201 formulation twice daily. Verhoeff‐VanGieson‐stained dermal tissue at 40× magnification.

Importantly, subjective assessments demonstrated strong concordance with blinded physician grading. At Week 12, 86% of subjects reported visible improvements in hydration, radiance, texture, and skin nourishment, while 72% perceived their skin as more youthful. High recommendation rates (86%) and reported optimism (90%) further support meaningful perceptual benefit. The alignment between objective measures and patient‐reported outcomes strengthens the translational significance of the intervention, indicating that biomechanical restoration translated into clinically perceptible improvement.

The temporal profile of response is also notable. Early improvements in firmness by Week 2 suggest rapid signaling effects, whereas cumulative biomechanical gains through Weeks 8–12 are consistent with progressive ECM remodeling. This pattern supports a model in which mTORC1 suppression first recalibrates intracellular stress‐response pathways, followed by structural reinforcement of the dermal matrix.

Selective mTORC1 modulation represents a differentiated strategy compared with traditional topical anti‐aging approaches. Many conventional agents stimulate fibroblast proliferation or collagen production through receptor‐mediated signaling, often without addressing upstream drivers of aging biology. In contrast, RLX‐201 targets a conserved longevity pathway that governs proteostasis and cellular maintenance. Unlike broad mTOR inhibition, which may suppress both mTORC1 and mTORC2 and potentially disrupt epidermal homeostasis, selective modulation of mTORC1 may allow restoration of autophagic balance while preserving essential anabolic signaling. This selectivity is particularly relevant for long‐term topical use.

From a broader longevity perspective, human skin offers a uniquely accessible model for studying tissue‐level aging. Mechanical properties such as elasticity and viscoelastic recovery function as quantifiable phenotypic readouts of underlying cellular health. Demonstrating that modulation of a canonical longevity pathway can improve these parameters provides proof‐of‐concept that healthspan‐oriented interventions can yield measurable benefits in human tissue.

Several limitations warrant consideration. The study duration was limited to 12 weeks, and longer‐term follow‐up is necessary to determine durability of biomechanical improvements. The clinical cohort included only female subjects, limiting generalizability. Molecular analyses performed in 3D human skin models should be interpreted as mechanistic support rather than direct in vivo confirmation. Future studies incorporating autophagy markers, mitochondrial functional assays, senescence markers, and transcriptomic profiling would further clarify the downstream consequences of selective mTORC1 modulation.

Despite these limitations, the convergence of molecular pathway suppression, histologic matrix reinforcement, objective biomechanical improvement, and concordant clinical and subject‐reported outcomes provides multi‐level evidence that RLX‐201 promotes restoration of functional skin resilience. Rather than delivering transient cosmetic enhancement, selective mTORC1 modulation appears to address a foundational driver of tissue aging dysregulated nutrient sensing, thereby supporting structural integrity and mechanical performance.

Collectively, these findings suggest that recalibrating conserved longevity pathways in human skin can translate into measurable improvements in tissue biomechanics and perceived function. This approach positions selective mTORC1 modulation as a promising strategy for extending skin healthspan and provides a translational framework for evaluating longevity‐targeted interventions in accessible human tissues.

## Author Contributions

Z.D.D., M.J., R.C.M., and M.K.R. conceived and designed the study. Z.D.D. conducted and executed the study. Z.D.D. and M.K.R. analyzed the data and performed the statistical analyses. M.K.R. wrote the manuscript. Z.D.D., M.J., R.C.M., and M.K.R. revised the final manuscript. All authors have read and approved the final manuscript.

## Ethics Statement

This study was conducted in accordance with the ethical principles of the Declaration of Helsinki (revised 2024). The study protocol was reviewed and approved by the Institutional Review Board (IRB) of Allendale Institutional Review Board (Protocol Approval Number: DCS‐100‐24; Date of Approval: 5/19/2025). All participants provided written informed consent prior to enrollment. Specific written consent was also obtained for the publication of identifiable clinical photographs in all formats.

## Consent

The authors confirm that written informed consent was obtained from all patients for the publication of their clinical images and case details within this article. The patients were informed of the worldwide, open‐access nature of the publication and have had the opportunity to review the manuscript and associated figures prior to submission. All identifying information has been removed to ensure patient anonymity.

## Conflicts of Interest

The authors declare no conflicts of interest.

## Data Availability

The datasets generated and/or analyzed during the current study are available from the corresponding author upon reasonable request. Certain data may be subject to confidentiality, intellectual property, or commercial restrictions.
